# Surgical castration with pain relief affects the health and productive performance of pigs in the suckling period

**DOI:** 10.1186/s40813-017-0066-1

**Published:** 2017-09-06

**Authors:** Joaquin Morales, Andre Dereu, Alberto Manso, Laura de Frutos, Carlos Piñeiro, Edgar G. Manzanilla, Niels Wuyts

**Affiliations:** 1 PigCHAMP Pro Europa S.L. c, Santa Catalina, 10, Segovia, Spain; 2 Zoetis Inc, Hoge Wei 10, 1930 Zaventem, Belgium; 3 Teagasc, Pig Development Department, Moorepark, Fermoy, Co Cork Ireland

**Keywords:** Boar breeding, Entire boars, Pre-weaning pig mortality, Stop castration, Suckling piglet, Surgical castration, Swine

## Abstract

**Background:**

Surgical castration is still practiced in many EU countries to avoid undesirable aggressive behavior and boar taint in male pigs. However, evidence shows that castration is painful and has a detrimental influence on pig health. This study investigated the clinical and productive effects of surgical castration in the suckling period.

A total of 3696 male pigs, 3 to 6 days old, comprising of 721 litters from two different farms were included in the study. Within each litter, half of the males were kept as intact males (IM) and half were surgically castrated (CM). Surgical castration was conducted by a trained farmer. Average daily gain (ADG), body weight at weaning (BWW), percentage of pre-weaning mortality (PWM) and antibiotic usage were measured. Pig major acute phase protein (PigMAP) serum concentrations were analyzed prior to castration, and on days 1 and 10 after castration. Productive performance data were analyzed using a linear mixed model. Mortality and percentage of pigs treated with antibiotics were analyzed using the Fisher’s exact test.

**Results:**

No overall differences in BWW and ADG were observed between the two groups. However, differences were observed when the same effects were analyzed in the 25% lightest, 50% medium and 25% heaviest pigs at birth. PWM was higher in CM than in IM groups (6.3% vs 3.6%; *p <* 0.001), especially in the light (12.2% vs 6.2%; *p =* 0.02) and in the medium (5.5% vs 2.7%; *p =* 0.04) weight groups. In the heaviest pigs group PWM was not affected by castration, but IM tended to show higher ADG (*p =* 0.06) and showed higher BWW (8.0 kg vs 7.8 kg; *p =* 0.05) than CM. There were no differences in percentage of pigs treated with antibiotics between the two groups (5.8% vs 5.8%; *p* = 0.98) in this study. Furthermore, PigMAP was increased in CM the day after castration (0.944 mg/ml vs 0.847 mg/ml; *p* = 0.025), but there was no difference between CM and IM groups at day 10.

**Conclusions:**

Surgical castration has a negative impact on production in the suckling period because it causes an increase in PWM, especially in pigs in the three lower quartiles for body weight, and negatively affects the BWW in pigs born in the highest quartile for body weight.

## Background

Intact male pigs have better feed conversion and can have higher growth rates than surgically castrated pigs (barrows) [1]. However, in many countries, male pigs are routinely castrated to prevent boar taint that results from the presence of androstenone or skatole [2], and also to reduce undesirable aggressive and sexual behaviour following the onset of puberty [3, 4].

Current European legislation allows surgical castration up to an age of 7 days of age [5]. However, as castration of pigs is also a substantial animal welfare problem, European agreements specified that from 2012 onwards physical castration of pigs should be performed with prolonged analgesia and/or anesthesia and that it should be abandoned totally by 2018 [6]. Available evidence shows that castration is both painful and stressful for the animal during and for some time after the castration [7, 8]. Potential complications associated with surgical castration include hemorrhage, excessive swelling or edema and infection: these can reduce performance, compromise health and, in some cases, increase mortality. A meta-analysis of 15 studies in 2009 showed that male piglets that had been surgically castrated had significantly higher mortality rates than their intact littermates [9]. However, existing literature provides little consistent information about the effects of surgical castration on the timing and causes of mortality, the incidence of different disease problems and on how the growth rate of the castrated pigs is affected in the suckling period.

This study aims to evaluate the clinical and productive effects of surgical castration with pain relief in the suckling period and to describe the different causes of mortality associated with this surgical procedure.

## Methods

### Animals, facilities and experimental design

This research was carried out at two one-site commercial swine herd farms, located in Segovia, Spain. Both farms were farrow-to-finish farms and were located in the same geographical area, had the same genetic lines and feed provider and similar size (630 and 570 reproductive sows in Farm 1 and Farm 2, respectively). The experimental design was a randomized block design, including surgical castration as the main effect, resulting in two experimental treatment groups with surgically castrated males (CM) and male pigs kept as intact males (IM).

Any sows with a clinical history of high incidence of abortions, high percentage of stillborn or high percentage of pre-weaning mortality were excluded from the study. At day 107 of gestation sows were moved from the gestation barn to farrowing pens. In both farms, each farrowing pen had a partially slatted floor and a heat bulb for the piglets. A blank creep feed was offered from day 14 of age to the piglets *ad libitum*. Following normal practice in both farms, teeth clipping and tail docking were performed on all piglets enrolled in this study before Study Day 0. Male pigs were individually identified by ear tagging, weighed and randomized on Study Day 0 (day 3 to 6 of life). Randomization was done within litter at individual animal level and based on body weight. A different randomization list was used for each litter. Directly after randomization piglets in CM group were surgically castrated. Cross-fostering was only allowed before Study Day 0. The study observations ended on weaning at 28 days of age of the piglets. Normal animal housing and management procedures usually employed on the farms were used throughout the experimental period and animals were managed in compliance with the European farm animal welfare regulations [5].

### Surgical castration procedure

A non-steroidal anti-inflammatory drug (NSAID) (meloxicam; 0.4 mg meloxicam/kg body weight, Contacera®: Zoetis) was administered 30 min before castration to mitigate pain, following recommendations proposed by O’Connor *et al*. [10]. The use of NSAIDs was not a common practice in the two farms selected, but the study aimed to represent the ‘best practice’ as it should be applied according to the 2012 European agreement [6]. Farm workers, previously trained by a veterinarian in order to standardise the procedure between farms, performed the castration according to normal farm practice between days 3 and 6 of life. In brief, piglets were restrained by the farmer workers. Two vertical incisions were made in the scrotum and the testes were removed after tearing off the spermatic cord. After castration, a topical antibiotic was administered on the injury area (oxytetracycline; Tenicol spray: MSD) for 5 s. Routine use of an antibiotic spray was used in this study in order to ensure optimal post-surgical recovering in the castrated group.

### Observations and measurements

A general physical examination was performed for all enrolled pigs on Study Day 0. Body weight was measured from all piglets on Study Day 0 (3–6 days of age) and at weaning (28 days of age), and average daily gain (ADG) in the study period was calculated. Daily general health observations were carried out by the farm workers and ill or injured piglets were promptly examined and treated by the veterinarian. For all treatments the following information was recorded: animal identification, date, product used, dose, frequency, as well as reason for treatment (the routine use of the post castration antibiotic spray was not recorded or analysed as a treatment in the study observation period). In addition, for any piglet found dead or euthanized on welfare grounds during the study, a necropsy was conducted and the reason for death was recorded. Piglets in very poor health were removed from the study and placed with a nurse sow to give them a chance to recover. All adverse health observations were recorded. The different reasons for mortality or removals were listed as deaths associated to complications following the surgical castration procedure, meningitis, diarrhoea, runt piglets and other causes that could be related to post-castration complications. Meningitis was assigned to piglets that showed nervous signs (leaning head, pedaling and convulsions) before death or sudden deaths. Diarrhoea was assigned to piglets showing liquid faeces, dehydration, and poor body condition before death. Runt piglets were piglets which showed poor body condition and cachexia as the only clinical signs before death. All other minor causes of death or removal (e.g. dermatitis) were classified as ‘other’.

Different reasons for antibiotic treatment interventions were classified as diarrhoea, dermatitis, runt piglets, meningitis, respiratory signs (coughing, rapid breathing, discharges from the eyes-conjunctivitis, sneezes, etc) and lameness (arthritis).

An acute phase-protein, PigMAP (Major Acute Phase-protein of Pigs), was used as an unspecific biomarker to quantify inflammation and/or stress. Fifty litters from Farm 1 and 40 litters from Farm 2 were randomly selected using a random number table for blood sampling. In each of these 90 litters, two piglets (1 CM and 1 IM) were also randomly selected (180 piglets in total; 100 in Farm 1 and 80 in Farm 2) and were blood sampled by venopunction of the vena cava on day 0 (before castration), day 1 (the day after castration) and day 10. Serum was immediately removed after centrifugation at 3500 g for 5 min, and kept frozen (−20 °C) until their analysis. PigMAP concentration in serum was measured by sandwich ELISA with two monoclonal antibodies, using a commercial kit (pigMAP kit ELISA, Acuvet biotech, Zaragoza, Spain) as described earlier [11].

### Statistical analysis

All treatment differences were assessed at the 2-sided 0.05 alpha level of significance and trends were reported for alpha = 0.10. In all cases of an interaction with *p* < 0.10, the interaction was studied. Multiple comparisons were adjusted using Tukey’s correction.

Piglets were classified based on their weight at day 0 in quartiles: light (25% lightest pigs), medium and heavy (25% heaviest pigs). The primary variables average weight at weaning and ADG were analyzed using a general linear model (proc GLM) including treatment, parity, farm and body weight group (light, medium, heavy) and all their second degree interactions as fixed factors.

PigMAP serum concentrations were analyzed using a linear mixed model (proc MIXED) with the fixed effects of treatment, parity, farm and second degree interactions effect.

Data on casualties and medication was analyzed using generalized linear models (proc GLIMMIX) including treatment, parity, farm, body weight group and all their second level interactions as fixed factors. When the frequency of events (casualties or medication) observed was 0 in any level of the independent variables (treatment, BW group or their interaction), the model did not converge, and the analysis was done using Fisher’s exact test.

All statistical analyses were carried out using SAS version 9.4.

## Results

A total of 3696 crossbred (Large White & Landrace x Pietrain) male pigs from 721 litters were included in the study. In Farm 1, a total of 1950 piglets from 363 litters and in Farm 2, 1746 piglets from 358 litters were included in the study. A total of 1848 piglets were assigned to each treatment group (IM, CM).

Productive performance (Table 1) showed an interaction between surgical castration and weight group (body weight at weaning, *p =* 0.084). Castration did not affect performance of light or medium piglets but heavy CM animals tended to have a lower ADG (*p* = 0.059) and had a lower body weight at weaning (*p =* 0.05). Pre-weaning mortality (Table 2) also showed an interaction between surgical castration and weight group (*p =* 0.063). Mortality was almost double for light (*p =* 0.017) and medium (*p =* 0.041) weight CM piglets but was not different for heavy CM animals (*p =* 0.327). No other variable or interaction affected productive performance or pre-weaning mortality.

**Table 1:** Effect of surgical castration on growth rate in the suckling period by initial body weight group^*a*^

Item	Intact Males	Castrated Males	*SEM* ^*b*^	*P value* ^*3*^
Light pigs				
Initial number of pigs	476	459		
Body weight, day 0 (kg)	1.43	1.42	0.008	0.528
Body weight, day 28 (kg)	5.69	5.77	0.062	0.367
Average daily gain (g/day)	182.3	185.5	2.648	0.379
Medium pigs				
Initial number of pigs	921	931		
Body weight, day 0 (kg)	1.96	1.97	0.006	0.226
Body weight, day 28 (kg)	6.83	6.79	0.041	0.540
Average daily gain (g/day)	210.0	208.5	1.768	0.535
Heavy pigs				
Initial number of pigs	451	458		
Body weight, day 0 (kg)	2.55	2.57	0.012	0.388
Body weight, day 28 (kg)	7.97	7.81	0.063	0.050
Average daily gain (g/day)	237.4	230.4	2.615	0.059

^a^Pigs were split in three groups according to their initial body weight: light pigs (25% pigs with the lightest initial body weight), medium pigs (50% pigs with medium initial body weight) and heavy pigs (25% pigs with the heaviest initial body weight)

^b^Standard Error of Mean

^3^ *P*-value for surgical castration treatment effect (Intact vs. Castrated male pigs) obtained by multiple comparisons using Tukey’s correction

**Table 2:** Effect of surgical castration on percentage of mortality in the suckling period by initial body weight group^*a*^

Item	Intact Males (%)	Castrated Males (%)	*Odds ratio* ^*b*^ *(95% CI)*	*P value* ^*3*^
Light	6.3	12.2	2.2 (1.09, 4.25)	0.017
Medium	2.7	5.5	2.1 (1.02, 4.22)	0.041
Heavy	2.4	1.5	0.6 (0.15, 2.51)	0.327

^a^Pigs were split in three groups according to their initial body weight: light pigs (25% pigs with the lightest initial body weight), medium pigs (50% pigs with medium initial body weight) and heavy pigs (25% pigs with the heaviest initial body weight)

^b^Odds ratio of death

^3^ *P*-value for surgical castration treatment effect (Intact vs. Castrated male pigs) obtained by multiple comparisons and using Tukey’s correction

**Fig. 1:**
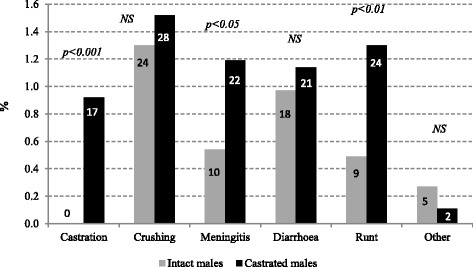
Different reasons of losses in intact and in castrated male pigs during the suckling period in percent. Number of cases is detailed in each column

The percentages for different causes of death are presented in Fig. 1. Percentage of mortality associated with the procedure of castration, with meningitis and losses of runt piglets were higher in CM than in IM group. In total 17 CM piglets died due to castration: 4 of them had a non-detected testicular hernia and died during the surgical procedure; 3 piglets died within 1 h after the surgical procedure showing an haemorrhage in the wound area and the other 10 died within 5 days after the procedure of castration showing an infection in the surgical area. No differences between CM and IM were observed in the other reasons of mortality or removal. Odds ratios for mortality were 2.3 (1.1, 4.8) for meningitis and 2.8 (1.3, 6.0) for runt pigs, when surgical castration was applied. Body weight group affected mortality associated to castration (0.75, 0.54 and 0.00% for light, medium and heavy piglets respectively; *p =* 0.021), crushing (2.78, 1.03 and 0.77%; *p =* 0.002), and meningitis (1.60, 0.76, 0.33%; *p =* 0.012).

No differences in antibiotic treatment due to castration were observed in this study. Highest frequency of antibiotic treatment was to treat diarrhoea (4.20%) followed by treatment of meningitis (0.98%). Body weight group affected antibiotic treatment for meningitis (2.99, 1.46 and 0.66% for light, medium and heavy piglets respectively; *p =* 0.002).

PigMAP serum concentration was significantly higher in CM than in IM groups the day after castration, and these differences disappeared by day 10 (Table 3).

**Table 3:** Effect of surgical castration on PigMAP^a^ serum concentration (mg/ml) at three different study days

Item	Intact Males	Castrated Males	*SEM* ^*b*^	*P value*
Day 0	0.903	0.841	0.032	0.167
Day 1	0.847	0.944	0.030	0.025
Day 10	0.990	0.902	0.073	0.392

^a^Pig major acute phase protein

^b^Standard Error of Mean

Day 0 = sampling before castration, day 1 = 1 day after castration, and day 10 = 10 days after castration in castrated males. In intact male piglets, the days correspond to the intervention in their litter mates

## Discussion

Castration is a surgical procedure commonly performed in non-optimal hygiene conditions on 3–6 days-old piglets and results in open wounds. Until now, very little research quantifying the health and performance impact of castration on male piglets, especially in the suckling period, has been published.

In the present study, castration was conducted under commercial conditions by two experienced farmers. In brief, meloxicam was administered about 30 min prior to castration in male pigs in CM group, and a topical antibiotic was applied on the injury wounds after castration. No other manipulations were imposed, to replicate commercial situations as much as possible. Under these conditions, surgical castration almost doubled the percentage of pre-weaning mortality, mainly associated with runting of affected piglets, with fatal meningitis, and with the intra- and the post-surgery mortality. Most studies evaluating the consequences of castration rarely mention pre-weaning mortality [12], suggesting that there is no effect. However, analysis of data from commercial herds shows that poor hygiene at castration could promote the occurrence of arthritis, which itself may result in death of piglets [13]. In another study, a lower antibody response to an immune challenge in castrated piglets compared to entire piglets was observed [14], probably attributable to the stress reaction, which could explain the higher mortality. In our study, PigMAP serum concentration was significantly higher in CM than in IM the day after castration, confirming that physical castration causes tissue damage, inflammation and stress. This result is in accordance with a previous study [15], where a significant increase of haptoglobin serum concentration, another acute phase protein, 24 h after physical castration was also observed, likely associated with stress or with an inflammatory process. Since in this study most antibiotic interventions were not specific to castration, it was not possible to make an assessment about antibiotic usage as a result of castration or non-castration. Furthermore, it should be noted that all piglets in the CM group received a topical antibiotic (spray) as a post-surgery prophylactic measure.

Castration reduces undesirable aggressive and sexual behaviors, but it also stimulates fat deposition and has a negative effect on feed conversion [12]. In another study, a decrease in the growth rate of piglets was observed only in the days following surgery when it was carried out shortly after birth (3 days) [16]. These differences in weight gain had disappeared by weaning, as was also observed in the present study, prompting the conclusion that castration does not have long-term implications in the suckling period. However, taking into account our observation that the mortality rate was almost double in CM group compared to the IM group, an alternative explanation is more likely: i.e. that there is a disproportional impact on weakest pigs (those with lowest bodyweight) in the CM group with higher mortality whilst in the IM group the survival rate of these low weight piglets was higher. The mean growth performance picture in the whole group obscures the effect of these opposite interactions.

Reduced health recovery may be related to less time nursing and more time lying down of castrated pigs [17]. Adequate colostrum intake and regular consumption of milk are required for control of gastrointestinal diseases, hypoglycemia, starvation, and crushing of the pre-weaned pig [18]. In addition, lightweight pigs are most susceptible to diseases [19, 20], which was confirmed by the results of the present study. This observation is especially important in the current status of the swine industry, where selection for maximum prolificacy has resulted in an increase in litter size and more piglets weaned per litter. This rapid increase in litter size has resulted in an increase of light-birth-weight piglets [21], thus exacerbating the (negative) effects of castration as a critical management parameter. Moreover, in the group of pigs with the heaviest birth weight, even if surgical castration did not result in higher mortality, it compromised growth rates to weaning. This observation could be associated with stress and discomfort promoted by the castration procedure, affecting milk consumption. As a consequence of both higher mortality rate in the light pigs and poorer growth performance in the heavy pigs, surgical castration applied in 3 to 6 days of age male piglets has a negative impact on health, performance and economic production cost in the suckling period.

## Conclusions

Surgical castration promotes productive losses in the suckling period by causing an increase in pre-weaning mortality in pigs born with the lowest body weights, and negatively affects growth rate and weaning body weight in pigs born with highest body weights. Main causes of mortality related to surgical castration were runt pigs, meningitis and the surgical and post-surgical complications of the castration procedure.

## Abbreviations

ADG: Average daily gain
BWW: Body weight at weaning
CM: Surgically castrated male pigs
IM: Intact male pigs
PigMAP: Pig major acute phase protein
PWM: Pre-weaning mortality

## References

[1] Bonneau M (1998). Use of entire males for pig meat in the European Union. Meat Sci.

[2] Zamaratskaia G, Squires EJ (2009). Biochemical, nutritional and genetic effects on boar taint in entire male pigs. Animal.

[3] Cronin GM, Dunshea FR, Butler KL, McCauly I, Barnett JL, Hemsworth PH (2010). The effects of immune- and surgical castration on the behaviour and consequently growth of group-housed, male finisher pigs. Appl Anim Behav Sci.

[4] Rydhmer L, Lundström K, Andersson K (2010). Inmunocastration reduces aggressive and sexual behaviour in male pigs. Animal.

[5] European Union. Council Directive 2008/120/EC of 18 December 2008 laying down minimum standards for the protection of pigs. OJ L 47, 18.2.2009.

[6] European Commission. European Declaration on alternatives to surgical castration of pigs. https://ec.europa.eu/food/sites/food/files/animals/docs/aw_prac_farm_pigs_cast-alt_declaration_en.pdf. Accessed 19 May 2017.

[7] Hay M, Vulin A, Genin S, Sales P, Prunier A (2003). Assessment of pain induced by castration in piglets: behavioral and physiological responses over the subsequent 5 days. Appl Anim Behav Sci.

[8] Kluivers-Poodt M, Houx BB, Robben SRM, Koop G, Lambooij E, Hellebrekers LJ (2012). Effects of a local anaesthetic and NSAID in castration of piglets, on the acute pain responses, growth and mortality. Animal.

[9] Allison J, Pearce M, Brock F, Crane JA. 2010. A comparison of mortality (animal withdrawal) rates in male fattening pigs reared using either physical castration or vaccination with Improvac® as the method to reduce boar taint. Proc. 21st IPVS Congress, Vancouver, Canada. July 18-21. 2010, p. 1139.

[10] O’Connor A, Anthony R, Bergamasco L, Coetzee J, Gould S, Johnson AK, Karriker LA, Marchant-Forde JN, Martineau GS, McKean J, Millman ST, Niekamp S, Pajor EA, Rutherford K, Sprague M, Sutherland M, von Borell E, Dzikamunhenga RS (2014). Pain management in the neonatal piglet during routine management procedures. Part 2: Grading the quality of evidence and the strength of recommendations. Anim Health Res Rev.

[11] Piñeiro M, Lampreave F, Alava MA (2009). Development and validation of an ELISA for the quantification of pig major acute phase protein (Pig-MAP). Vet Immunol Immunop.

[12] Prunier A, Bonneau M, von Borell EH, Cinotti S, Gunn M, Fredriksen B, Giersing M, Morton DB, Tuyttens FAM, Velarde A (2006). A review of the welfare consequences of surgical castration in piglets and the evaluation of non-surgical methods. Anim Welf.

[13] Strom I (1996). Arthritis in piglets. Dansk Veterinaertidsskrift.

[14] Lessard M, Taylor AA, Braithwaite L, Weary DM (2002). Humoral and cellular immune responses of piglets after castration at different ages. Can J Anim Sci.

[15] Lackner A, Goller K, Ritzmann M, Heinritzi K. 2002. Acute phase proteins in castration of piglets. Proc. 17th IPVS Congress, Ames, IA, USA. June 2-5. 2002, Vol 2 p. 253.

[16] Kielly J, Dewey CE, Cochran M (1999). Castration at 3 days of age temporarily slows growth of pigs. Swine Health Prod.

[17] McGlone JJ, Nicholson RI, Hellman JM, Herzog DN (1993). The development of pain in young pigs associated with castration and attempts to prevent castration: Induced behavioral changes. J Anim Sci.

[18] Roy B, Kumar A, Lakhani GP, Jain A (2014). Causes of pre-weaning mortality in India. Schol J Agric Sci.

[19] Beaulieu AD, Aalhus JL, Williams N, Patience JF (2010). Impact of piglet birth weight, birth order, and litter size on subsequent growth performance, carcass quality, muscle composition, and eating quality of pork. J Anim Sci.

[20] Fix JS, Cassady JP, Holl JW, Herring WO, Cusbertson MS, See MT (2010). Effect of piglets birth weight on survival and quality of commercial market swine. Livest Sci.

[21] Bérard J, Kreuzer M, Bee G (2008). Effect of litter size and birth weight on growth, carcass and pork quality, and their relationship to postmortem proteolysis. J Anim Sci.

